# Evaluation of Bioactive Compounds and Chemical Elements in Herbs: Effectiveness of Choline Chloride-Based Deep Eutectic Solvents in Ultrasound-Assisted Extraction

**DOI:** 10.3390/molecules30020368

**Published:** 2025-01-17

**Authors:** Aleksandra Szydłowska-Czerniak, Agnieszka Kowaluk, Michał Strzelec, Tomasz Sawicki, Małgorzata Tańska

**Affiliations:** 1 Department of Analytical Chemistry and Applied Spectroscopy, Faculty of Chemistry, Nicolaus Copernicus University in Toruń, 87-100 Toruń, Poland; 2 Central Office of Measures, Laboratory of Electrochemical and Inorganic Analyzes, Department of Physical and Environmental Chemistry, 00-139 Warszawa, Poland; agnieszka.kowaluk@gum.gov.pl (A.K.); michal.strzelec@gum.gov.pl (M.S.); 3 Department of Human Nutrition, Faculty of Food Science, University of Warmia and Mazury in Olsztyn, 10-718 Olsztyn, Poland; tomasz.sawicki@uwm.edu.pl; 4 Department of Food Plant Chemistry and Processing, Faculty of Food Science, University of Warmia and Mazury in Olsztyn, 10-718 Olsztyn, Poland

**Keywords:** herbs, eco-friendly solvents, ultrasound-assisted extraction, antioxidant capacity, antioxidant compounds, multi-elemental profile, ultra-performance liquid chromatography, inductively coupled plasma–mass spectrometry

## Abstract

In this study, the effectiveness of three choline chloride (ChCl)-based deep eutectic solvents (DESs) formed using malonic acid (MalA), glycerol (Gly), and glucose (Glu) as hydrogen bond donors and two conventional solvents (50% methanol and 50% ethanol) for ultrasonic-assisted extraction (UAE) of antioxidant compounds from four herbs (chamomile, lemon balm, nettle, and spearmint) were estimated. The antioxidant capacity (AC) of the obtained herb extracts was determined by the modified 2,2′-azino-bis(3-ethylbenzothiazoline-6-sulfonic acid) (ABTS), 2,2-diphenyl-1-picrylhydrazyl (DPPH), and cupric reducing antioxidant capacity (CUPRAC) methods. Profiles of phenolic acids, flavonoid aglycones, and flavonoid glycosides in the green and conventional herb extracts were quantitatively analyzed using ultra-performance liquid chromatography (UPLC). Among the prepared DESs, the highest antioxidant potential and total contents of phenolic acids, flavonoid aglycones, and flavonoid glycosides in herb extracts were achieved using ChCl:MalA (1:1). Unexpectedly, the selected green solvents extracted significantly lower amounts of total antioxidants from the investigated herbs than 50% alcohols. Additionally, macroelements (K, Na, Ca, Mg), micronutrients (Mn, Zn, Fe, Cu), and a toxic element (Cd) in four herbs were analyzed using inductively coupled plasma–mass spectrometry (ICP–MS). Determining the compositions of antioxidants and elements in herbs is essential for understanding their nutritive importance when applied in the food, cosmetic, and pharmaceutical industries.

## 1. Introduction

Herbs have been used since ancient times for both culinary purposes, enhancing the flavor, aroma, and color of food and beverages, and medicinal purposes, providing protection against acute and chronic diseases and supporting overall health [1]. Herbal medicine has consistently been recognized as a fundamental component of primary healthcare [2,3]. According to the World Health Organization (WHO), approximately 80% of the global population is estimated to rely on herbal medicinal products for their therapeutic benefits [4].

The literature data indicate that herbs exhibit antioxidant, anti-inflammatory, anti-cancer, anti-carcinogenic, and glucose- and cholesterol-lowering effects, as well as properties that influence cognitive function and mood [1,5,6,7]. These health-promoting properties are attributed to the high content of numerous bioactive compounds present in herbs, such as phenolic acids, flavonoids, terpenoids, essential oils, alkaloids, coumarins, saponins, tannins, vitamins, minerals, organosulfur compounds, and polysaccharides [1,8]. However, the availability and efficacy of herb bioactive compounds for therapeutic or nutritional applications are significantly influenced by the technique and solvent used for their extraction.

Conventional extraction procedures (i.e., maceration, infusion, steam or hydro-distillation, decoction, percolation, and Soxhlet extraction) are quite laborious, time-consuming, and involve large amounts of organic solvents such as hydrocarbons, alcohols, and chloroalkanes, raising environmental and health concerns [9]. Several novel approaches are effectively employed for the extraction of bioactive compounds from plants, including ultrasound-, microwave-, infrared-, and cold plasma-assisted extractions; super- and subcritical fluid extractions; pulsed electric field extraction; pressurized liquid extraction; and enzyme-assisted extraction [10,11,12,13,14]. Among them, ultrasound-assisted extraction (UAE) offers several advantages over other modern extraction techniques. It is faster, requires less energy, and is compatible with a wide range of solvents. Additionally, scaling up UAE for industrial applications is straightforward to and involves lower equipment and operational costs, making it accessible to both small laboratories and large-scale industries. These attributes have contributed to its growing popularity across various sectors [14]. A key advantage of UAE is its ability to preserve the bioactive properties of extracts, since it utilizes ultrasonic cavitation, a process involving the formation, expansion, and collapse of microbubbles in a solvent that breaks down cell walls and facilitates the release of intracellular compounds [15,16].

In the past decade, new environmentally friendly “green” solvents such as deep eutectic solvents (DESs) have been considered for the extraction of bioactive compounds from plants [9,17,18]. These solvents, especially those composed of choline chloride (ChCl) as the hydrogen bond acceptor (HBA) and hydrogen bond donors (HBD) such as carboxylic acids, alcohols, and carbohydrates, offer unique physicochemical properties, low toxicity, and biodegradability and can be easily tailored for specific applications [19,20]. The literature data indicate that ChCl-based DESs have been effectively employed for the extraction of phenolic compounds from plant tissues, plant-based products, and biomass [21,22,23,24]. However, their application in herbal analysis remains underexplored and requires further investigation. Moreover, studies on the extraction of bioactive compounds from herbs using DESs have predominantly focused on comparing extraction yields of total phenolic content and evaluating the antioxidant properties of the extracts. Far less attention has been focused on understanding the impact of DESs on the microstructure and elemental composition of herbs post-extraction.

It is well known that different bioactive compounds and their antioxidant capacities make various herbs of interest for food, cosmetics, and medical applications. In addition, many macro- and microelements found in herbs are essential for human health, and their extraction could potentially enhance the nutritional quality of the resulting extracts [25,26,27]. Apart from the beneficial properties of antioxidants and minerals present in herb plants, there might be a hidden health risk due to the presence of trace toxic metals in them. Notably, the genetic background of plants, including herbs, affects the content of secondary metabolites such as flavonoids and the uptake and accumulation of metals [28,29]. Some authors have found a correlation between metals and flavonoids, such as enhancing the transportation of flavonoids within plants through their complexation with metals [29,30]. Moreover, flavonoid–metal complexes were considerably more effective radical scavengers than free flavonoids. The increase in the antioxidant activity of metal–flavonoid compounds depends on the central metal ion, the flavonoid type used, and the molar ratio of metal to flavonoid [30].

Therefore, advanced and modified analytical methods are essential to ensure the overall quality of herbal products. Combined studies of organic analytes, such as bioactive compounds, and inorganic analytes, including element profiles, provide a deeper understanding of the composition of herbs.

In this context, our study aimed to evaluate, for the first time, the antioxidant capacity (AC), involving various mechanisms, and the phenolic compound profiles of extracts from four herbs (chamomile, lemon balm, nettle, and spearmint) obtained using ultrasonic-assisted extraction (UAE) combined with choline chloride-based DESs formulated with different HBDs, including malonic acid (MalA), glycerol (Gly), and glucose (Glu). Additionally, the microstructure of the herbs post-extraction as well as the localization and content of chemical elements in the herbs before and after extraction were examined using green spearmint as a representative example. Inductively coupled plasma mass spectrometry (ICP-MS) was employed to analyze variations in the elemental composition of the herb samples. A comparative analysis of the effectiveness of DESs relative to conventional solvents (50% methanol and 50% ethanol) highlighted both the advantages and limitations of these DESs.

## 2. Results and Discussion

### 2.1. Antioxidant Capacity of Herb Extracts

Three hydrophilic DESs, easily formed from choline chloride as an inexpensive, non-toxic, biodegradable small quaternary ammonium salt, and HBDs, representant of carboxylic acids (malonic acid), alcohols (glycerol), and carbohydrates (glucose), were applied as green solvents for UAE of antioxidants from chamomile, lemon balm, nettle, and spearmint. Under the same conditions, antioxidants were extracted from these four herbs using two conventional solvents (50% methanol and 50% ethanol).

The AC results of chamomile, lemon balm, nettle, and spearmint extracts determined by the modified 2,2′-azino-bis(3-ethylbenzothiazoline-6-sulfonic acid) (ABTS), 2,2-diphenyl-1-picrylhydrazyl (DPPH), and cupric reducing antioxidant capacity (CUPRAC) assays are presented in Table 1. The Shapiro–Wilk test indicated that all AC data for each herb prepared in different solvents is normally distributed (having a *p*-value greater than 0.05, *p* = 0.0893–0.9056, *p* = 0.1905–0.9484, and *p* = 0.0820–0.9711 for ABTS, DPPH, and CUPRAC results, respectively). Moreover, the Brown–Forsythe test confirmed that variances among AC of herb extracts prepared using different solvents were homogeneous (*p* = 0.2907–0.7618, *p* = 0.1157–0.2969, and *p* = 0.1656–0.5141 for ABTS, DPPH, and CUPRAC results, respectively). Therefore, significances were analyzed by one-way ANOVA with the Duncan post hoc test for multiple comparisons of the AC means of herb extracts prepared in different solvents.

As can be seen, the ABTS, DPPH, and CUPRAC values for herb extracts prepared using green and traditional solvents differ significantly (Table 1, Duncan test). Unexpectedly, methanolic (ABTS = 261.9–2432.0 μmol TE/g, DPPH = 79.2–502.2 μmol TE/g, CUPRAC = 109.6–505.2 μmol TE/g) and ethanolic extracts (ABTS = 204.5–2006.9 μmol TE/g, DPPH = 93.4–510.4 μmol TE/g, CUPRAC = 99.6–548.7 μmol TE/g) of the investigated herbs revealed significantly higher antioxidant capacity determined by three different analytical methods than ABTS (6.5–672.2 μmol TE/g), DPPH (0.00–101.8 μmol TE/g), and CUPRAC (0.00–155.8 μmol TE/g) values for samples after extraction by three DESs. Regardless of the analytical method, methanolic and ethanolic extracts of lemon balm had the highest AC results (ABTS = 2432.0 and 2006.9 μmol TE/g, DPPH = 502.2 and 510.4 μmol TE/g, and CUPRAC = 505.2 and 548.7 μmol TE/g, respectively), whereas all studied extracts of nettle had the lowest antioxidant potential (ABTS = 6.5–261.9 μmol TE/g, DPPH = 0.0–93.4 μmol TE/g, and CUPRAC = 0.00–109.6 μmol TE/g). Interestingly, the Duncan test indicated that DPPH values for alcoholic chamomile and lemon balm extracts did not differ significantly (Table 1).

Similarly, in our previous research, the DPPH (15.2–1244.9 μmol TE/g), ABTS (79.8–3887.6 μmol TE/g), and FRAP (8.7–461.4 μmol TE/g) values of the ChCl-based extracts of cinnamon, nutmeg, clove, ginger, cardamom, and coriander were significantly lower than the AC of the pure ethanolic (DPPH = 49.2–1754.3 μmol TE/g, ABTS = 184.8–10,350.3 μmol TE/g, FRAP = 28.1–1385.1 μmol TE/g) and 70% ethanolic (DPPH = 51.1–2199.5 μmol TE/g, ABTS = 145.5–7928.2 μmol TE/g, FRAP = 22.2–1030.8 μmol TE/g) extracts of these spices [31]. Moreover, Siamandoura and Tzia [32] found higher DPPH results for olive leaf extracts prepared using high hydrostatic pressure-assisted extraction (HHPAE) and two conventional solvents, such as water (IC_50_ = 4.0 g d.w./g DPPH) and 70% ethanol (IC_50_ = 8.0 g d.w./g DPPH) than those extracted using DESs composed of ChCl with maltose, glycerol, citric acid, and lactic acid (IC_50_ = 5.0–41.0 g d.w./g DPPH). Moreover, olive leaf ethanolic extracts (IC_50_ = 2.7 g d.w./g DPPH) obtained through homogenate-assisted extraction had higher DPPH radical scavenging activity compared to DPPH values of extracts prepared using DESs (IC_50_ = 4.0–8.4 g d.w./g DPPH).

Notably, the strong acid DES1 (ChCl:MalA) displayed remarkable effectiveness for the extraction of total antioxidants from spearmint (ABTS = 672.2 μmol TE/g, DPPH = 101.8 μmol TE/g, CUPRAC = 155.8 μmol TE/g), while weak acids DES2 (ChCl:Gly) and DES3 (ChCl:Glu) exhibited the highest AC values for chamomile (ABTS = 46.2 and 50.3 μmol TE/g, DPPH = 17.1 and 10.4 μmol TE/g, and CUPRAC = 19.4 and 18.2 μmol TE/g). The type of hydrogen bond donors (HBDs) in the studied ChCl-based DESs had an important effect on the acidity of DES mixtures and the efficacy extraction of antioxidant compounds from four herbs. It is noteworthy that strongly acidic DES1 created by combining ChCl and MalA at the molar ratio (1:1) extracted bioactive compounds from all herbs with the highest ability to scavenge ABTS radical cation, DPPH radical, and redox reaction and form Cu(I)-chelate. This can be explained by the fact that the presence of free hydrogen ions in the malonic acid contributes to the hydrolysis of the macromolecules of herb matrices, increasing the extractability of antioxidant compounds [24].

It is important to note that there were no significant differences between ABTS (except nettle extracts), DPPH (except chamomile extracts), and CUPRAC values of the studied extracts prepared using two weakly acidic DESs by combining ChCl with glycerol (DES2) and glucose (DES3) in a 1:2 and 2:1 molar ratio, respectively (Table 1, Duncan test). However, the acidity of DES2 containing glycerol was higher than that of DES3 with glucose, which provided a better ability to extract antioxidants from the investigated herbs (Table 1).

It is probable that the hydroxyl and carboxyl groups present in ChCl-based DES1 increased its polarity, hence facilitating the hydrogen bond interaction with the biomolecules. Although ChCl-based DES2 and DES3 have three and four OH groups, their performances in antioxidant extraction were significantly lower than ChCl-based DES1 due to a lack of synergistic effect between other functional groups, such as the carboxyl group. Moreover, the hydroxyl group position and the ChCl-to-HBD molar ratio affect the polarity and viscosity of ChCl-based DESs and, consequently, the extractability of antioxidants [33,34]. The highest number of hydroxyl groups of glucose surrounding the negatively charged chloride anion of ChCl probably decreased the polarity of the DES3, causing the lowest antioxidant extraction.

Similar effects of polarity, solubility, viscosity, surface tension, and physicochemical interactions of various DESs on the antioxidant properties of herbs were reported previously [35,36]. Betaine:urea (1:1) was the best DES to recover the antioxidants from chamomile, lemon balm, nettle, and spearmint able to generate a color Cu(I)-chelate (CUPRAC = 50–425 μmol TE/g), whereas only lemon balm and spearmint prepared in this DES had the highest ABTS values (about 140 μmol TE/g) [35]. However, the highest DPPH results (100–170 μmol TE/g) for all studied herbs were obtained using glucose and urea as DES after adding 50% water. Although the lower water content (30%) in this DES improved the extraction of antioxidants from chamomile and nettle, which can scavenge ABTS radical cation (83 and 138 μmol TE/g). Among the four DESs used by Koraqi et al. [36] for extraction of bioactive compounds from nettle before the DPPH determination, citric acid:maltose (DPPH = 1071.05 μmol TE/mL) was the most effective green solvent, followed by lactic acid:glucose (DPPH = 942.89 μmol TE/mL), citric acid:mannitol (DPPH = 750.51 μmol TE/mL), and citric acid:menthol (DPPH = 678.86 μmol TE/mL). Other authors proposed lactic acid-based DESs (lactic acid:choline chloride (3:1), lactic acid:sodium acetate (3:1), lactic acid:ammonium acetate (3:1), and lactic acid:glycine:water (3:1:3)) for ultrasound-assisted extraction of antioxidants from native Greek medicinal plants [37]. The spearmint extract obtained with the last DES exhibited significantly stronger reducing power (900 μmol ascorbic acid equivalents (AAE)/g d.w.) and the antiradical activity (2050 μmol DPPH/g d.w.) measured by FRAP and DPPH methods, respectively. Furthermore, among six ChCl-based DESs (ChCl: glycerol (1:1), ChCl:sorbitol (1:1), ChCl:malic acid (1:1), ChCl:citric acid (1:1), and ChCl:fructose (1:1) and (1:2)) served as solvents for the extraction of bioactive compounds from the *Mentha piperita* leaves, the highest potential to reduce ferric (Fe^3+^) to ferrous ion (Fe^2+^) (FRAP = 45.70 mg AAE/g d.w.) was observed with ChCl:sorbitol (1:1), while ChCl:malic acid (1:1) had the highest scavenging effect toward DPPH radical (IC_50_ = 0.67 mg/mL) [38]. Nevertheless, lemon balm extracts obtained with ChCl:1,2-propanediol (1:1), and ChCl:citric acid (1:1) had the most potent ability to neutralize DPPH radical (IC_50_ was about 0.20 mg/mL) and to reduce Fe^3+^ to Fe^2+^ (FRAP was above 6 mg AAE/mL), while lemon balm extract prepared in ChCl:urea (1:1) demonstrated the highest peroxyl radical scavenging capacity (ORAC = 140 μmol TE/mL) [39]. In contrast, ChCl:fructose (1:1) was the least effective for the recovery of bioactive compounds from lemon balm, which were able to scavenge DPPH radical (IC_50_ = 0.30 mg/mL) and peroxyl radical (ORAC = 75 μmol TE/mL), and donate electrons to oxidants and stabilize them (FRAP = 3.3 mg AAE/mL).

### 2.2. Phenolic Profiles of Herb Extracts

The phenolic compounds in extracts prepared from chamomile, lemon balm, nettle, and spearmint were investigated by determining the levels of phenolic acids and flavonoids. The results of the chromatographic analysis are presented in Table 2 and Table 3. The results indicated that, regardless of the type of herb, the extractability of phenolic compounds was significantly higher when using aqueous solutions of methanol and ethanol than DESs.

The extracts obtained with alcoholic solvents contained between 757.0 mg/100 g to 7028 mg/100 g of total phenolic compounds (Table 2). The extracts from lemon balm were the richest in phenolic compounds, with a total phenolic content (TPC) of approximately 7000 mg/100 g. Among the extraction solvents, the 50% methanol proved to be most effective. High extraction efficiency was also observed for spearmint extracts, with an average TPC of 6085 mg/100 g. In contrast, the alcoholic extracts from chamomile and nettle exhibited TPC values more than six times lower than those from lemon balm. However, a notable solvent effect on extraction efficiency was observed for nettle, where the TPC for the 50% methanol extract was 1.7 times higher than for the 50% ethanol extract. For the other herbs, the solvent choice had a minimal effect, with methanolic extracts showing TPC values 2–4% higher than ethanolic extracts. Most of the extracts were predominantly composed of phenolic acids, which accounted for between 68.4% of the TPC in the methanolic extracts from nettle and 94.7% in the methanolic extracts from lemon balm. The exception was the nettle extract prepared with 50% ethanol, where flavonoids dominated, representing 63.0% of the TPC, with aglycones making up 68.5% and glycosides 31.5%.

The proportions of individual phenolic acids in the alcoholic extracts mainly depended on the type of herb from which they were obtained. In the chamomile extracts, three phenolic acids—chlorogenic acid, ellagic acid, and rosmarinic acid—dominated, accounting for 22–29% of the total phenolic acids (TPA) regardless of the solvent used. A higher amount of ferulic acid was also present in these extracts, but its concentration in the extract obtained with 50% methanol was twice as high as in the extract prepared with 50% ethanol. The use of 50% methanol also resulted in the extraction of a significantly greater amount (over 2.5 times) of vanillic acid from chamomile compared to the use of 50% ethanol. Differences in extractability were also observed for gentisic acid, which was extracted using 50% ethanol at a concentration of 20.2 mg/100 g, while 50% methanol did not extract this phenolic acid from chamomile.

The phenolic acid profiles of the lemon balm and spearmint extracts obtained using aqueous solutions of both alcohols were found to be similar. In the lemon balm extracts, rosmarinic acid dominated, with concentrations of 5546 mg/100 g (50% methanol) and 5799 mg/100 g (50% ethanol), accounting for 83.3% and 90.3% of TPA, respectively. The type of alcohol used had a greater effect only on the content of ferulic acid in these extracts. The ferulic acid concentration in the lemon balm extracts obtained with 50% methanol was 500.0 mg/100 g, while in the extracts prepared with 50% ethanol, it was only 34.4 mg/100 g. Furthermore, the extraction using 50% methanol from spearmint allowed the extraction of 375.0 mg/100 g of this phenolic acid, which was an eightfold difference compared to the extract obtained with 50% ethanol.

However, in the case of nettle, not only TPA but also the proportions of individual phenolic acids were strongly influenced by the type of alcohol used for extraction. In the extract prepared with 50% methanol, ferulic acid dominated (655.0 mg/100 g), accounting for as much as 73% of TPA. This extract also contained a higher amount of chlorogenic acid, with a concentration of 93.0 mg/100 g. In contrast, the concentration of other phenolic acids in the methanolic extract of nettle did not exceed 5% of TPA. On the other hand, 50% ethanol was ineffective in extracting ferulic acid and chlorogenic acid from this herb, with concentrations 21.2 and 1.4 times lower, respectively, compared to the extraction with 50% methanol.

Compared to traditional solvents, three green solvents used in this study (ChCl:MalA (1:1), ChCl:Gly (1:2), and ChCl:Glu (2:1)) extracted only specific phenolic acids, with ex-traction efficiency depending on both the type of herb and the DES type employed (Table 3). Among the DESs analyzed, DES1 proved to be the most effective, extracting the highest amounts of gallic acid from chamomile and nettle (249.0 and 232.0 mg/100 g, respectively), and the highest amounts of rosmarinic acid from lemon balm and spearmint (259.0 and 461.0 mg/100 g, respectively). Spearmint extracts obtained using this solvent contained the highest overall concentrations of phenolic acids (TPA = 786.0 mg/100 g). In the spearmint extract prepared in DES1, notable amounts of ellagic acid (221.0 mg/100 g) were observed, while gentisic (37.8 mg/100 g), caffeic (29.8 mg/100 g), ferulic (15.8 mg/100 g), salicylic (14.3 mg/100 g), and syringic acids (3.8 mg/100 g) were present in lower concentrations. DES1 also extracted rosmarinic, ellagic, chlorogenic, ferulic, and syringic acids from chamomile, but their concentrations ranged between 2.4 and 39.2 mg/100 g. In lemon balm extracts obtained with DES1, higher amounts of gallic acid (151.0 mg/100 g) were observed, along with small amounts of chlorogenic, ellagic, and salicylic acids (up to 22.4 mg/100 g). The nettle extract also contained rosmarinic, chlorogenic, and ellagic acids, with concentrations ranging from 13.9 to 20.3 mg/100 g.

DES2 and DES3 most effectively extracted rosmarinic acid across all tested herbs, with concentrations in the extracts ranging from 19.2 to 59.6 mg/100 g. However, no phenolic acids were detected in the nettle extract prepared using DES3. Interestingly, unlike DES1, the other green solvents showed no capacity to extract gallic acid. Some extracts obtained with DES2 and DES3 contained small amounts of ellagic acid (in chamomile and spearmint extracts), syringic acid (in chamomile extracts), and chlorogenic acid (in chamomile extract with DES2), but their concentrations did not exceed 16 mg/100 g.

The total flavonoids (sum aglycones and glycosides) in extracts prepared using traditional solvents (50% methanol, 50% ethanol) ranged from approximately 330 mg/100 g in chamomile extracts to 1045 mg/100 g in spearmint extract prepared with 50% ethanol (Table 2). In all extracts, aglycones were predominant, accounting for 56.2–71.7% of the total flavonoid content (TFL). Among these compounds, apigenin and kaempferol were dominant in chamomile extracts (together comprising 40–45% of TFL, depending on the alcohol used), kaempferol and myricetin in lemon balm extracts (25% of TFL), rutin in nettle extracts (averaging 40% of TFL), and rutin and kaempferol in spearmint extracts (41–46% of TFL, depending on the alcohol type).

Among glycosides, kaempferol derivatives were the most abundant in chamomile extracts (28% of TFL). In lemon balm extracts, quercetin-3-O-glucoside, myricetin-O-rutinoside, and kaempferol-3-O-rutinoside constituted 27–32% of TFL, depending on the alcohol type. In spearmint extracts, kaempferol-3-O-rutinoside contributed 18–22% of TFL, depending on the alcohol used. In nettle extracts, quercetin-3-O-glucoside, myricetin-O-rutinoside, kaempferol-3-O-rutinoside, and isorhamnetin-3-O-rutinoside were present in comparable amounts, each constituting approximately 8–9% of TFL.

The application of green solvents had a variable impact on the flavonoid extractability from the studied herbs. For chamomile, the type of DES used had no significant effect on the flavonoid profile. Aglycones, mainly naringenin, quercetin, and kaempferol, were predominant, with contents ranging from 15.1 to 23.6 mg/100 g. Kaempferol-O-glucoside was also present in higher amounts (approximately 18 mg/100 g) in chamomile extracts prepared by all proposed types of DESs. The use of DES1, in contrast to other DESs, additionally extracted kaempferol-3-O-rutinoside in the amount of 18.0 mg/100 g. For lemon balm, this solvent allowed for the extraction of comparable amounts of quercetin, kaempferol, and quercetin-3-O-glucoside (each approximately 18 mg/100 g), as well as rutin (about 5 mg/100 g). Other DESs proved less effective for flavonoid extraction from lemon balm; only DES3 extracted 17.7 mg/100 g of quercetin. Nettle extracts obtained using green solvents contained only rutin and quercetin, with kaempferol only present in DES1-based extract. For spearmint, DES1 proved to be the most effective flavonoid solvent, extracting 207.3 mg of TFL per 100 g of herb, whereas other DESs extracted half as much of these compounds. This extract predominantly contained kaempferol-3-O-rutinoside, kaempferol, and rutin, which together accounted for 55% of TFL.

When comparing traditional and green solvents, it can be concluded that the ChCl-based DES with malonic acid as the HBD was more effective at extracting phenolic compounds from all studied herbs. However, its efficiency was significantly lower than that of conventional solvents, particularly 50% methanol. The highest percentage of phenolic compounds extracted by this solvent were from chamomile and nettle, achieving 42.5% and 25.4% efficiency, respectively, compared to 50% methanol. The extraction efficiency of phenolic compounds using the other DESs was low, with total phenolic content (TPC) levels ranging from 1% for lemon balm to 12.4% for chamomile, relative to those obtained with 50% methanol. Furthermore, flavonoids were generally easier to extract with DESs than phenolic acids, particularly quercetin and naringenin, whose extraction efficiencies compared to 50% methanol were 46.1–51.5% and 42.7–48.7%, respectively. Interestingly, DES2 and DES3 were ineffective at extracting phenolic acids. Rosmarinic acid was determined in DES2- and DES3-based extracts of all studied herbs. However, only chamomile extracts prepared using DES2 and DES3 contained approximately 16 mg/100 g of ferulic acid, corresponding to 15% efficiency compared to extraction with 50% methanol. Surprisingly, it was observed that DES1 extracted gallic acid in significant amounts from chamomile, lemon balm, and nettle (151.0–249.0 mg/100 g), whereas conventional solvents failed to extract this phenolic acid (Table 2 and Table 3).

Methanol, ethanol, and their combinations with water in various ratios are the most commonly used solvents for extracting phenolic compounds from plants due to their ability to dissolve a wide range of both polar and non-polar compounds [40]. In our study, we confirmed the significantly higher efficiency of these solvents in extracting phenolic compounds from herbs compared to ChCl-based DESs. However, numerous studies indicate that DESs may be more suitable for extracting these compounds. For instance, El Kantar et al. [41] demonstrated that DESs exhibited high extraction efficiency for polyphenols from grapefruit peel, providing a greener alternative to conventional solvents with a higher yield of the flavonoid naringin. Similarly, Choudhary et al. [42] found that ChCl-based DESs (formulated with water and glucose, fructose, xylose, glycerol, or malic acid) were more effective at extracting phenolic compounds from medicinal plants (*Ocimum sanctum*, *Terminalia bellerica*, and *Terminalia chebula*) than methanol. Notably, ChCl:malic acid:water was the most effective DES. The superior ability of ChCl:malic acid DES for extracting phenolic compounds, compared to other DESs used in studies, may be attributed to its higher acidity [43]. It is well-established that acidic media generally enhance the extraction of phenolic compounds, especially flavonoids [44]. Gómez-Urios et al. [45] confirmed that ChCl:malic acid and ChCl:glycerol were the most effective DESs for extracting bioactive compounds from orange peel compared to other DESs (acid-, sugar-, and polyalcohol-based). The DESs outperformed 50% ethanol in extracting 4-hydroxybenzoic acid, vanillic acid, chlorogenic acid, ferulic acid, catechin, p-coumaric acid, and caffeic acid. Kalyniukova et al. [46] demonstrated that DESs based on ChCl and fructose achieved higher extraction efficiency for polyphenols from Norway spruce roots compared to methanol, ethanol, and water. Meanwhile, Wojeicchowski et al. [47] found that aqueous DESs yielded higher extraction results for phenolic compounds from rosemary leaves compared to 70% ethanol, though lower than pure ethanol. The authors attributed this to the higher viscosity of DESs, particularly in their pure form. Lazović et al. [48] reported comparable levels of phenolic compounds in extracts from *Agrimonia eupatoria* when using methanol and a ChCl:glycerol mixture (1:1). Like the findings of our study, they noted that this DES was more efficient at extracting flavonoids than phenolic acids compared to alcoholic solvents. Roby et al. [49] found, as in our study, that methanol-based extracts contained higher levels of phenolic compounds than ethanol-based extracts. Choudhary et al. [42] highlighted that the yield of phenolic compounds is influenced not only by the type of solvent but also by the physical and chemical characteristics of samples. The same trend was also confirmed in this study.

### 2.3. Morphological Characterization and Element Localization in Spearmint

To explore the preliminary extraction mechanism of bioactive compounds from the studied herbs, scanning electron microscopy and dispersive energy spectroscopy (EDS) were used to observe the morphology and identify the presence of chemical elements only in spearmint due to DES1-based extract of this herb was the richest source of antioxidants.

#### 2.3.1. Scanning Electron Microscopic Analysis

**Figure 1:**
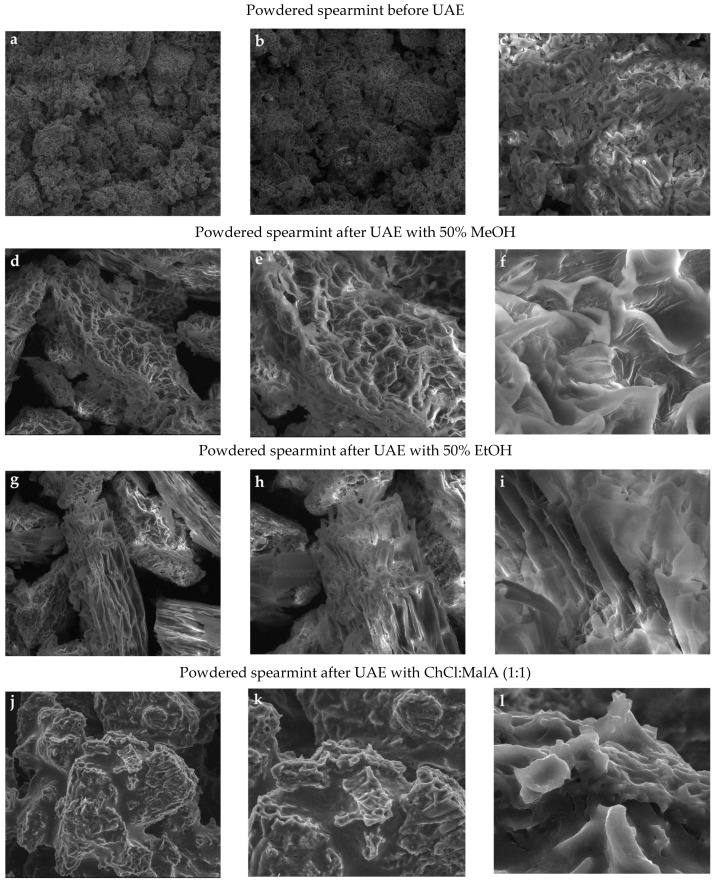
Scanning electron micrographs of spearmint powder before (**a**–**c**) and after ultrasound-assisted extraction with 50% methanol (**d**–**f**), 50% ethanol (**g**–**i**), choline chloride:malonic acid (1:1) (**j**–**l**) at 500× (**a**,**d**,**g**,**j**), 1000× (**b**,**e**,**h**,**k**), and 5000× (**c**,**f**,**i**,**l**) magnification, respectively.

The different magnification of the SEM was applied to closely observe the changes in texture, porosity, and any damage of spearmint cells caused by the extraction solvents and ultrasound waves during the UAE technique. The microstructural analysis of spearmint before and after UAE with 50% methanol, 50% ethanol, and ChCl:MalA (1:1) revealed clear variations in surface morphology using 500×, 1000×, and 5000× magnifications (Figure 1).

**Figure 2:**
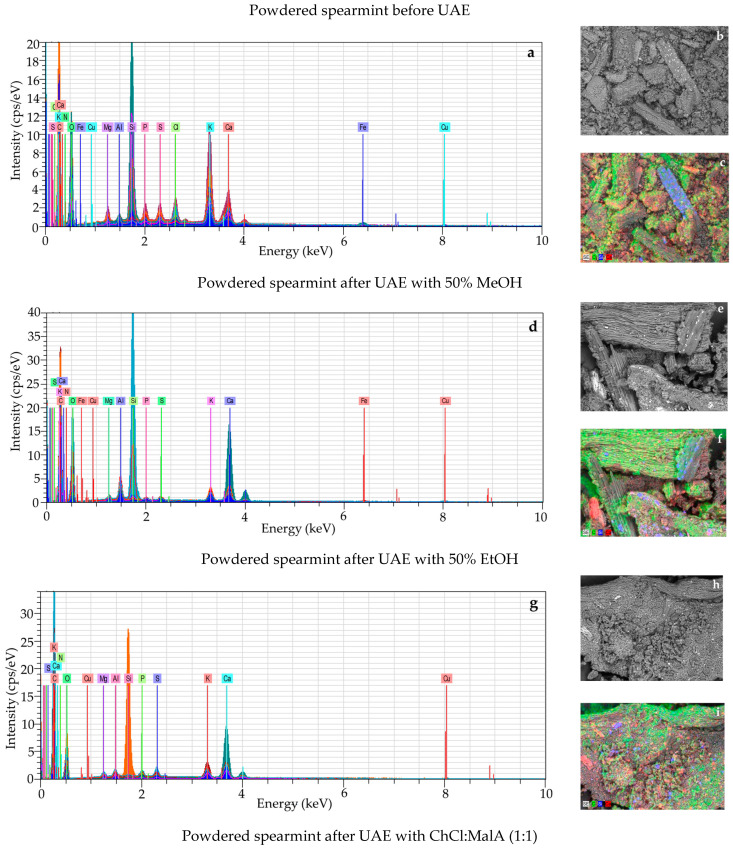

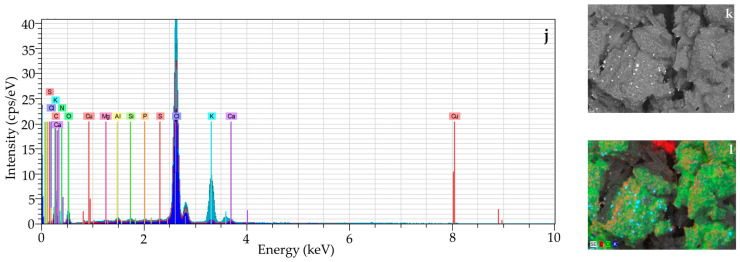
EDS spectra (**a**,**d**,**g**,**j**), SEM images (**b**,**e**,**h**,**k**), and mapping images via SEM–EDS (**c**,**f**,**i**,**l**) of spearmint powder before and after ultrasound-assisted extraction with 50% methanol, 50% ethanol, and choline chloride:malonic acid (1:1).

The structure of powdered original spearmint before the UAE exhibited a compact, dense mass with a smooth and relatively flat surface (Figure 2a–c). After the sonication process, the SEM images (Figure 2d–l) revealed a reduction in overall density, fragmentation, erosion, and detexturation. Moreover, a noticeable increase in tiny pores and cracks indicates that the solvents successfully penetrated and disrupted the structure of the solid residue. Importantly, the surface of the spearmint cells treated with ChCl:MalA (1:1) (Figure 2j–l) appeared to be more damaged than those after extraction with both conventional solvents such as 50% methanol (Figure 2d–f) and 50% ethanol (Figure 2g–i). As can be seen, the DES-UAE considerably destroyed the structural surface of the investigated solid residue, causing large pores, holes, cracks, and rough stacked fragments to be depicted. This can be explained by the fact that DESs can destroy the cell wall, whereas conventional solvents such as alcohols dissolve the bioactive compounds from the cell wall or permeate membranes to separate the individual cellular components.

Similarly, different ChCl-based DES such as ChCl:levulinic acid (1:2), ChCl:malic acid (1:1), ChCl:1,2-propylene glycol (1:2), ChCl:urea (1:3), and ChCl:lactic acid (1:2) destroyed the cell walls of various plant materials (leaves from *Celtis sinensis*, *Eucommia ulmoides* Oliver, broccoli, *Pluchea indica*, and black mulberry fruit powder), and drastic morphological changes in the surfaces of the solid residues based on SEM analysis were reported by other authors [50,51,52,53,54].

#### 2.3.2. Energy Dispersive X-Ray Spectroscopic Analysis

Energy dispersive spectroscopy (EDS) coupled with the SEM was applied to identify the presence of chemical elements in powdered spearmint before and after treatment of two conventional solvents (50% methanol and 50% ethanol) and one DES1 (ChCl:MalA (1:1)) (Figure 2).

The EDS spectra of untreated samples (Figure 2a) and those treated with conventional solvents (Figure 2d,g) indicated noticeable peaks and similar amounts of oxygen (66.45–70.08%), carbon (13.63–16.98%), and nitrogen (2.05–5.32%) elements, representative of the standard structure of natural plants containing oxygen functional groups such as carbonyl, hydroxyl, and others. On the contrary, the solid residue after DES-UAE (Figure 2j) contained significantly lower amounts of oxygen (51.59%) and carbon (8.42%) because MalA as HBD combined with the cavitation effect can partially disintegrate cellulose from spearmint cell walls and lead to the release of large levels of target compounds into the extract [50].

However, the EDS spectrum of the post-extraction residue with ChCl:MalA (1:1) showed 11.80% nitrogen and 21.51% chloride, confirming that ChCl, as a quaternary ammonium salt, is a rich source of nitrogen and chloride. Besides, chloride distribution in low amounts (0.16–1.59%) was only found for untreated spearmint (Figure 2a).

Moreover, signals from sulfur, phosphorus, magnesium, calcium, potassium, and copper were identified in the EDS spectra of all investigated samples (Figure 2a,d,g,j). Although Ca (3.32–4.99%) and Cu (0.27–0.35%) contents were the highest in residues after alcohol treatment and the lowest (Ca = 0.56% and Cu = 0.12%) in sample after DES extraction. In contrast, conventional solvents had a higher efficiency of potassium extraction. Therefore, its concentration (K = 1.51–1.55%) in the residues after extraction with 50% methanol and 50% ethanol was two times lower than in the residue after DES extraction (K = 3.54%) and in the initial spearmint sample (K = 3.46%). Interestingly, Fe (0.18%) was the most abundant in untreated spearmint amongst inorganic elements, and only 0.06% of Fe was identified in solid residual after 50% methanol treatment. This suggests that the chosen ChCl:MalA (1:1) and 50% ethanol demonstrated a high potential for iron recovery from various compounds in the plant material.

Minute traces of Si (0.32%) were found in the ChCl:MalA (1:1) pretreated sample, while significantly higher Si concentration in spearmint residues before (3.11%) and after extraction with alcohols (1.91–3.94%) was observed. In addition, the appearance of Al in all spectra could be due to the aluminum stub on which adhesive tape with the powder sample adhered. On the other hand, the EDS mapping images of samples treated with alcohols and DES describe the presence of uniformly dispersed C, Si, Ca and C, Cl, and K in the structures, respectively.

For comparison, EDS spectra of untreated kenaf fiber and kenaf fiber pretreated with lactic acid:ChCl (2:1) at biomass-to-DES ratios of 1:12 and 1:15 revealed the presence of only oxygen and carbon elements [55].

### 2.4. Elemental Compositions of Herbs

The concentrations of four macroelements (K, Na, Ca, Mg), four micronutrients (Mn, Zn, Fe, Cu), and a toxic element (Cd) in four studied herbs were analyzed using the ICP-MS method, and the obtained results are listed in Table 4.

It is noted that three macroelements, potassium, calcium, and magnesium, were determined at the highest amounts (K = 16,597–24,979 mg/kg, Ca = 7065–32,926 mg/kg, and Mg = 1461–5354 mg/kg), while sodium content (Na = 37.3–843 mg/kg) was significantly lower in all investigated herbs. Interestingly, spearmint showed the highest concentrations of potassium (K = 24,979 mg/kg) and magnesium (Mg = 5354 mg/kg), whereas nettle and chamomile were the richest sources of calcium (Ca = 32,926 mg/kg) and sodium (Na = 84.3 mg/kg), respectively. Moreover, the Duncan test indicated that lemon balm and nettle as well as lemon balm and spearmint did not differ significantly in amounts of potassium and calcium, respectively (Table 4). Among the studied essential elements, iron contents (Fe = 105–495 mg/kg) were the highest in all herbs, whereas over 20 times lower amounts of copper (Cu = 4.5–13.6 mg/kg) were determined. It is evident that spearmint revealed the highest levels of manganese (Mn = 152 mg/kg) and zinc (Zn = 31.7 mg/kg), but the lowest concentrations of all investigated micronutrients (4.5–105 mg/kg) were measured in chamomile. Insignificant differences in Zn and Cu concentrations were observed between lemon balm and spearmint as well as nettle and spearmint samples, respectively (Table 4, Duncan test). These results indicate that spearmint was rich in some micronutrients necessary for human health due to their acting in various physiological and enzymatic processes [56].

On the other hand, cadmium, which exhibits toxic and detrimental effects on the human body, was present in lemon balm, nettle, and spearmint at significantly lower doses (0.046–0.088 mg/kg) than the limit recommended by the WHO and FAO for vegetables (the maximum permissible value of Cd is 0.2 mg/kg of vegetable) [57]. Unfortunately, the Cd content (Cd = 0.506 mg/kg) in chamomile was more than two times higher than the prescribed limit. Therefore, chamomile contained the lowest macro- (except sodium content) and micronutrients but the highest toxic cadmium amount among the studied herbs. Specifically, the determined cadmium concentration in chamomile can be related to environmental conditions (natural contamination, bioaccumulation from the soil, pollution).

For comparison, Suliburska and Kaczmarek [26] found that the spearmint and nettle samples available on the Polish market had similar contents of minerals, including Mg (4330–5150 mg/kg and 4170–4940 mg/kg), Zn (22.1–28.0 mg/kg and 18.4–25.9 mg/kg), Fe (354–1080 mg/kg and 495–665 mg/kg), and Cu (14.4–17.2 mg/kg and 14.0–16.3 mg/kg) to these element levels observed in the studied herbs (Table 4). However, lower calcium concentrations (11,030–12,540 mg/kg and 13,240–14,950 mg/kg) in spearmint and nettle were determined using the atomic absorption spectrometry (AAS) method after mineralization. On the contrary, the chamomile flowers contained significantly higher amounts of essential microelements, including Zn (24.7–33.1 mg/kg), Fe (274–1020 mg/kg), and Cu (14.3–26.5 mg/kg). Although calcium content was similar (Ca = 5690–7520 mg/kg), a more than twofold higher magnesium level (Mg = 3020–3320 mg/kg) was reported by these authors in chamomile [26]. Additionally, the element profile (Na = 134.0–545.0 mg/kg, K = 19,400–49,500 mg/kg, Ca = 13,700–20,000 mg/kg, Mg = 2967–4529 mg/kg, Mn = 36.6–117.0 mg/kg, Zn = 26.1–142 mg/kg, Fe = 339–2080 mg/kg) found for *Mentha longifolia,* collected in different zones in North Ossetia and determined using neutron activation analysis at the IBR-2 reactor, did not differ significantly in comparison with element contents in our spearmint sample [27]. Importantly, similar results of micronutrients were observed for *Melissa officinalis* (Mn = 86 mg/kg, Zn = 25.1 mg/kg, Fe = 187 mg/kg, Cu = 8.20 mg/kg) and *Mentha piperita* (Mn = 26.1–118 mg/kg, Zn = 18.6–25.0 mg/kg, Fe = 147–407 mg/kg, Cu = 6.88–11.6 mg/kg) extracts prepared using a natural deep eutectic solvent (ChCl:oxalic acid) during microwave-assisted extraction and analyzed by inductively coupled plasma-optical emission spectrometry (ICP OES) [58]. In the case of poisonous cadmium, its concentration was one order higher than that recorded in our study (Table 4), amounting to 0.44 mg/kg in *Melissa officinalis* and ranging from 0.24 to 0.37 mg/kg in *Mentha piperita*.

One possible explanation for these discrepancies between the concentrations of evaluated elements in various herb samples is their botanical origin, climatic and cultivation conditions, soil composition and fertilization, environmental pollution, the technological processes applied to the raw material, and storage conditions.

## 3. Materials and Methods

### 3.1. Reagents

All analytical or HPLC grade reagents needed for the preparation of DESs and analyses of antioxidant capacity, as well as profiles of phenolic compounds and chemical elements, were purchased from Merck Sp. z o. o. (Warszawa, Poland).

### 3.2. Plant Materials

Four herbs (dried aerial parts of chamomile, lemon balm, nettle, and spearmint) were kindly obtained from a local company in the original polypropylene packaging. Immediately before extract preparation, each herb was pulverised in a laboratory mill (FW100 model, Chemland, Stargard, Poland) to give powders with approximate mean particle diameter (approximately 0.5 mm).

### 3.3. Preparation of Choline Chloride-Based Deep Eutectic Solvents

Green solutions were synthesized using a heating method. Three ChCl-based DESs were prepared by heating different molar ratios (1:1, 1:2, and 2:1) of ChCl and the various HBDs, such as malonic acid (MalA), glycerol (Gly), and glucose (Glu) at 80 °C under constant stirring until clear liquids were produced (about 120 min). Additionally, 20% water was added to each DES to decrease viscosity and facilitate extraction.

The synthesized DESs were kept in sealed glass bottles in the dark at ambient temperature. The codes of the DESs used in this study were as follows: DES1— ChCl:MalA (1:1), DES2 – ChCl:Gly (1:2), and DES3— ChCl:Glu (2:1).

### 3.4. Ultrasound-Assisted Extraction of Bioactive Compounds

The UAE was carried out according to a previously described methodology [35] using a Sono Swiss SW 6H ultrasound bath (Labo Plus, Warszawa, Poland) operating at an ultrasound power of 540 W and an ultrasound frequency of 37 kHz at a temperature of 50 °C for 4 min. First, 0.5 g of each ground and sieved (0.5 mm) herb were weighed precisely on an analytical balance, and 5 mL of each DES with 20% water or conventional solvents (50% methanol and 50% ethanol) were added and treated for ultrasonication. The same herb sample was extracted in triplicate. After the extraction, DES-based and conventional extracts were centrifuged for 5 min at 4500 rpm using a laboratory centrifuge (MPW-150R, MPW MED. INSTRUMENTS, Warszawa, Poland). The obtained supernatants were placed in capped bottles and stored in a refrigerator at 4 °C until the antioxidant properties were analyzed.

### 3.5. Analytical Methods

#### 3.5.1. Spectrophotometric Determination of Antioxidant Capacity

The antioxidant properties of herb extracts prepared using three DESs and two conventional solvents were determined by using three analytical tests that measured antiradical activity against ABTS radical cation and DPPH radical (ABTS and DPPH radical scavenging activity assays, respectively) and the reducing capacity of Cu(II)-neocuproine complex (CUPRAC assay) by the extracted bioactive compounds. These procedures were described in detail previously [35]. The absorbance of the obtained color solutions was immediately read using a Hitachi U-2900 spectrophotometer (Tokyo, Japan), and the calculated AC was expressed as μmol Trolox equivalent (TE) per g of each herb sample in all analytical assays.

#### 3.5.2. Chromatographic Determination of Bioactive Compounds

The qualitative and quantitative analysis of phenolic compounds in the prepared herb extracts was performed using an Ultra-High Performance Liquid Chromatography (UHPLC) system (Nexera XR, Shimadzu, Kyoto, Japan) coupled with a diode area detector (DAD) and mass spectrometer (LCMS-2020, Shimadzu, Kyoto, Japan), following the methodology described by Sawicki et al. [59] and Chociej et al. [60]. The phenolic compounds were separated using a C18 BEH column (1.7 μm particle size, 2.1 mm (diameter) × 100 mm (length); Waters, Warszawa, Poland). The eluent consisted of 0.01% formic acid in water with 2 mM ammonium formate (A) and 0.01% formic acid in 95% acetonitrile with 2 mM ammonium formate (B), with a flow rate of 0.15 mL/min. The oven temperature was set to 50 °C, and the sample injection volume was 10 µL. Scanning was performed in negative ionization mode, and the analysis was conducted in selected ion monitoring (SIM) mode. The compounds were identified based on their characteristic ions, retention times, and λ_max_ values, as reported in the previously published data [59,60]. Quantification of the phenolic compounds was performed by correlating the UHPLC-DAD-MS peak area with commercially available standards.

#### 3.5.3. Surface Morphology Analysis by Scanning Electron Microscopy with an Energy Dispersive X-Ray Spectrometer

Concentrated samples were dehydrated using a vacuum freeze dryer (Alpha 1–2 LDplus, Martin Christ, Osterode am Harz, Germany) and frozen in liquid nitrogen before measurements.

The microstructure of untreated and extracted spearmint by 50% methanol, 50% ethanol, and ChCl:MalA (1:1) was examined by scanning electron microscopy/focused ion beam (SEM/FIB) using a Quanta 3D FEG microscope (Carl Zeiss, Göttingen, Germany). Additionally, the morphology and elemental composition of these samples were analyzed with SEM LEO Electron Microscopy Ltd., 1430 VP (Cambridge, United Kingdom) equipped with detectors of backscattered electrons (BSE), cathodoluminescence (CL), and an energy dispersive X-ray spectrometer (EDS) Quantax with an XFlash 4010 detector (Bruker AXS microanalysis GmbH, Berlin, Germany).

#### 3.5.4. Determination of Element Concentrations

Before analysis of elements, powdered herbs (0.2000 g) were placed in the closed Teflon vessel and digested with 6 mL diluted nitric acid (1:1 *v*/*v*) and 2 mL of hydrogen peroxide (30%) in a closed Anton Paar Multiwave Pro microwave system in an 8NXF100 rotor (Anton Paar GmbH, Graz, Austria). The conditions in the microwave digestion program for four mineralization vessels included two stages: (1st) 15 min of power up to 800 W, and (2nd) 40 min at a constant power of 800 W, max temperature 200 °C. After digestion, the samples were diluted to 50 mL with deionized water. A corresponding blank was also prepared according to the above microwave-assisted digestion procedure. Each digested sample was introduced into the inductively coupled plasma mass spectrometer (ICP-MS Agilent 7800, Agilent Technologies, Inc., Tokyo, Japan) via a crossflow nebulizer, spray chamber, and autosampler. The operating parameters of the ICP-MS spectrometer used in the determination of elements are given in Table 5.

Multi-element calibration solutions for instrument calibration were prepared by diluting single-element mass concentration standards of the Central Office of Measures.

### 3.6. Statistical Analysis

The determinations of the AC, phenolic contents, and element concentrations in the investigated herb samples were carried out in threefold, duplicate, and sixfold, respectively, and the values obtained were averaged and presented as a mean ± standard deviation (SD). The Shapiro–Wilk normality test and Brown–Forsythe variance homogeneity test were used to analyze the sample distribution. The data of the normally distributed assays were compared with one-way ANOVA and the significance of the differences was analyzed with Duncan multiple range test, with the significant value being *p* < 0.05. Statistical analysis was performed with the help of Statistica 8.0 software (StatSoft, Tulsa, OK, USA).

## 4. Conclusions

Three DESs based on choline chloride (HBA) and malonic acid (MalA), glycerol (Gly), and glucose (Glu) (HBDs) in different molar proportions were synthesized as promising green solvents for ultrasound-assisted extraction of bioactive compounds from four herb samples. Although the proposed DESs were less efficient for antioxidant recovery from the studied herbs (chamomile, lemon balm, nettle, and spearmint) than conventional solvents (50% methanol and 50% ethanol), they are superior to commonly used alcohols in extraction processes due to their low cost, non-toxicity and biocompatibility. These results show that in the prepared ChCl-based DESs, different HBDs interact with target bioactive compounds to affect their extraction efficiency. Therefore, DES composed of ChCl and MalA (1:1) revealed the highest efficiency for extraction of total phenolic acids, flavonoid aglycones, and flavonoid glycosides. Moreover, spearmint extract obtained by ChCl:MalA–UAE exhibited the highest AC regardless of the three analytical methods applied (ABTS, DPPH, and CUPRAC), while alcoholic extracts of lemon balm were the richest sources of bioactive compounds with the highest antioxidant potential.

Additionally, the synergistic influence of ultrasonication and solvent type on the element composition and morphology of spearmint tissues was analyzed and compared using SEM and EDS. This approach allowed the assessment of the effectiveness of green ChCl:MalA (1:1) and conventional solvents for extracting not only antioxidants but also various elements based on the element compositions of spearmint residues after the UAE process. On the other hand, the health benefits of the investigated herbs are mostly related to mineral content. These studies confirm that analyzed herbal materials were potential sources of minerals. Macro- (K, Na, Ca, Mg) and microelements (Mn, Zn, Fe, Cu) in the investigated herbs using ICP-MS spectrometry were determined in concentrations indicating possible influence on pharmacological action regarding their therapeutic application. Notably, the contents of toxic cadmium in lemon balm, nettle, and spearmint were below the WHO limit, whereas the highest Cd concentration above the recommended value (0.20 mg/kg) was found in chamomile.

The obtained results can help improve the efficiency in selecting ChCl-based DESs for the extraction of bioactive compounds from herbs according to their properties, which could be potentially used at an industrial scale as green, effective, and sustainable solvents. It is worth emphasizing that the extraction efficiency of individual phenolic compounds depends not only on the type of solvent used but also on the type of raw material. Therefore, the extraction process for bioactive compounds should be carefully optimized for each specific material prior to extraction. This highlights the necessity of modifying extraction methods in future studies to maximize efficiency and ensure the selective recovery of specific compounds in herbal extracts.

## Figures and Tables

**Table 1:** Antioxidant capacity (AC) of herbal extracts obtained using three DESs and 50% methanol and 50% ethanol.

Herb	AC * ± SD (μmol TE/g)				
	DES1	DES2	DES3	50% MeOH	50% EtOH
ABTS method					
Chamomile	174.0 ± 7.1 ^b^	46.2 ± 1.8 ^a^	50.3 ± 0.8 ^a^	338.4 ± 9.2 ^c^	380.1 ± 5.6 ^d^
Lemon Balm	320.2 ± 12.6 ^b^	47.7 ± 0.5 ^a^	24.0 ± 0.7 ^a^	2432.0 ± 44.5 ^d^	2006.9 ± 83.5 ^c^
Nettle	88.8 ± 4.3 ^c^	16.4 ± 0.6 ^b^	6.5 ± 0.3 ^a^	261.9 ± 7.2 ^e^	204.5 ± 7.4 ^d^
Spearmint	672.2 ± 3.2 ^b^	37.0 ± 0.6 ^a^	46.8 ± 2.1 ^a^	1116.1 ± 38.2 ^c^	1566.8 ± 58.2 ^d^
DPPH method					
Chamomile	39.6 ± 1.7 ^c^	17.1 ± 0.6 ^b^	10.4 ± 0.3 ^a^	102.6 ± 1.3 ^d^	103.0 ± 4.2 ^d^
Lemon Balm	50.0 ± 0.8 ^b^	13.8 ± 0.6 ^a^	4.0 ± 0.1 ^a^	502.2 ± 20.8 ^c^	510.4 ± 16.5 ^c^
Nettle	40.3 ± 1.3 ^b^	1.8 ± 0.1 ^a^	<DL	79.2 ± 2.0 ^c^	93.4 ± 2.9 ^d^
Spearmint	101.8 ± 2.9 ^b^	11.3 ± 0.4 ^a^	11.7 ± 0.5 ^a^	287.2 ± 11.7 ^c^	413.5 ± 14.6 ^d^
CUPRAC method					
Chamomile	25.8 ± 1.2 ^b^	19.4 ± 0.2 ^a^	18.2 ± 0.9 ^a^	128.4 ± 6.3 ^c^	135.5 ± 4.1 ^d^
Lemon Balm	40.1 ± 1.2 ^b^	16.5 ± 0.2 ^a^	8.4 ± 0.4 ^a^	505.2 ± 19.1 ^c^	548.7 ± 10.5 ^d^
Nettle	15.1 ± 0.6 ^b^	5.6 ± 0.2 ^a^	<DL	109.6 ± 4.7 ^d^	99.6 ± 3.7 ^c^
Spearmint	155.8 ± 1.6 ^b^	15.5 ± 0.04 ^a^	15.6 ± 0.5 ^a^	316.1 ± 1.4 ^c^	349.4 ± 11.4 ^d^

* *n* = 3; SD—standard deviation; DES1—choline chloride:malonic acid (ChCl:MalA) (1:1); DES2—choline chloride:glycerol (ChCl:Gly) (1:2); DES3—choline chloride:glucose (ChCl:Glu) (2:1); 50% MeOH—50% methanol; 50% EtOH—50% ethanol; DL—detection limit; Different letters within the same row (a–e) indicate significant differences between the antioxidant capacity of herb extracts prepared by various solvents and determined using the same analytical method (one-way ANOVA and Duncan test, *p* < 0.05).

**Table 2:** Phenolic profiles of herbal extracts obtained using 50% methanol and 50% ethanol (results expressed in mg/100 g).

Herb	Chamomile		Lemon Balm		Nettle		Spearmint	
	50% MeOH	50% EtOH	50% MeOH	50% EtOH	50% MeOH	50% EtOH	50% MeOH	50% EtOH
Benzoic acid	40.7 ± 0.1 ^c^	61.6 ± 2.8 ^f^	38.2 ± 0.7 ^b^	28.2 ± 0.7 ^a^	39.2 ± 0.2 ^b,c^	42.4 ± 1.0 ^d^	50.3 ± 0.6 ^e^	50.8 ± 0.8 ^e^
Caffeic acid	0 ± 0 ^a^	0 ± 0 ^a^	142.0 ± 2.4 ^d^	143.0 ± 1 ^d^	0 ± 0 ^a^	0 ± 0 ^a^	74.8 ± 0.4 ^b^	78.4 ± 0.5 ^c^
Chlorogenic acid	235.5 ± 2.1 ^h^	176.1 ± 2.0 ^g^	7.8 ± 0.1 ^a^	9.9 ± 0.1 ^b^	93.0 ± 2.1 ^f^	67.7 ± 0.5 ^e^	59.5 ± 1.2 ^d^	53.9 ± 1.0 ^c^
p-Coumaric acid	20.1 ± 0.0 ^a^	22.0 ± 0.1 ^b^	22.4 ± 0.1 ^b,c^	22.5 ± 0.0 ^c^	20.1 ± 0.1 ^a^	20.3 ± 0.0 ^a^	26.3 ± 0.4 ^d^	26.5 ± 0.8 ^d^
Ellagic acid	187.0 ± 5.0 ^d^	197.0 ± 5.0 ^e^	65.3 ± 0.8 ^c^	61.6 ± 0.4 ^c^	17.4 ± 0.5 ^a^	55.7 ± 1.0 ^b^	2342 ± 62 ^f^	2599 ± 53 ^g^
Ferulic acid	102.0 ± 6.0 ^e^	50.3 ± 0.5 ^d^	500.0 ± 2.0 ^g^	34.4 ± 0.1 ^b^	655.0 ± 29.0 ^h^	30.9 ± 0.0 ^a^	375.0 ± 9.0 ^f^	46.8 ± 0.2 ^c^
Gallic acid	0 ± 0 ^a^	0 ± 0 ^a^	0 ± 0 ^a^	0 ± 0 ^a^	0 ± 0 ^a^	0 ± 0 ^a^	0 ± 0 ^a^	0 ± 0 ^a^
Gentisic acid	0 ± 0 ^a^	20.2 ± 0.2 ^b^	46.5 ± 1.7 ^c^	48.2 ± 0.6 ^c^	0 ± 0 ^a^	0 ± 0 ^a^	168.0 ± 2.0 ^e^	52.5 ± 1.0 ^d^
Rosmarinic acid	180.0 ± 2.0 ^c^	194.0 ± 1.0 ^d^	5546 ± 170 ^g^	5799 ± 29 ^h^	41.1 ± 0.1 ^b^	39.4 ± 0.1 ^a^	1921 ± 24 ^e^	2054 ± 9 ^f^
Salicylic acid	15.1 ± 0.1 ^b^	14.6 ± 0.2 ^a^	247.2 ± 4.6 ^g^	228.4 ± 3.0 ^f^	25.2 ± 0.6 ^c^	23.8 ± 0.3 ^c^	144.0 ± 2.1 ^e^	130.6 ± 2.6 ^d^
Syringic acid	4.6 ± 0.2 ^b^	5.9 ± 0.2 ^c^	35.3 ± 0.5 ^e^	37.4 ± 1.7 ^e^	0 ± 0 ^a^	0 ± 0 ^a^	5.9 ± 0.4 ^c^	12.9 ± 0.2 ^d^
Vanillic acid	17.1 ± 1 ^g^	6.6 ± 0.0 ^d^	5.3 ± 0.0 ^c^	7.5 ± 0.1 ^e^	5.0 ± 0.1 ^b^	0 ± 0 ^a^	10.7 ± 0.0 ^f^	5.0 ± 0 ^b^
TPA	803.0 ± 16.0 ^c^	748.0 ± 4.0 ^b^	6655 ± 173 ^h^	6420 ± 29 ^g^	896.0 ± 31.0 ^d^	280.0 ± 1.0 ^a^	5179 ± 101 ^f^	5111 ± 67 ^e^
Apigenin	58.9 ± 0.9 ^e^	76.4 ± 2.9 ^g^	4.5 ± 0.0 ^a^	5.3 ± 0.0 ^c^	4.9 ± 0.0 ^b^	4.8 ± 0.0 ^b^	56.4 ± 0.6 ^d^	74.3 ± 0.7 ^f^
(+)-Catechin	13.1 ± 0.0 ^b^	0 ± 0 ^a^	26.8 ± 0.2 ^b^	27.3 ± 0.1 ^b^	30.2 ± 0.1 ^d^	29.4 ± 0.2 ^c^	31.7 ± 0.1 ^e^	31.5 ± 0.4 ^e^
Kaempferol	71.1 ± 1.1 ^e^	74.2 ± 0.2 ^f^	52.2 ± 0.4 ^c^	66.1 ± 0.4 ^d^	0 ± 0 ^a^	36.0 ± 0 ^b^	216.2 ± 1.6 ^g^	244.0 ± 2.0 ^h^
Myricetin	0 ± 0 ^a^	0 ± 0 ^a^	43.6 ± 0.3 ^c^	43.5 ± 0.3 ^c^	0 ± 0 ^a^	0 ± 0 ^a^	0 ± 0 ^a^	37.5 ± 0.0 ^b^
Naringenin	31.4 ± 0.0 ^b^	31.5 ± 0.0 ^b^	31.4 ± 0.0 ^b^	31.6 ± 0.0 ^b^	30.2 ± 0.0 ^a^	31.3 ± 0.0 ^b^	35.4 ± 0.1 ^c^	37.2 ± 0.1 ^d^
Quercetin	37.5 ± 0.1 ^d^	0 ± 0 ^a^	35.7 ± 0.1 ^c^	39.7 ± 0.1 ^e^	35.2 ± 0.0 ^b^	41.1 ± 0.0 ^f^	0 ± 0 ^a^	37.5 ± 0.1 ^d^
Rutin	24.0 ± 0.7 ^b^	26.3 ± 0.2 ^c^	21.9 ± 0.1 ^a^	28.7 ± 0.0 ^d^	167.0 ± 4.0 ^e^	184.4 ± 1.9 ^f^	170.0 ± 4.0 ^e^	184.1 ± 2.4 ^f^
TAG	235.9 ± 0.9 ^c^	208.4 ± 3.3 ^a^	216.0 ± 0.1 ^b^	242.3 ± 0.1 ^d^	268.0 ± 4.0 ^e^	327.0 ± 2.0 ^f^	510.0 ± 4.0 ^g^	646.0 ± 6.0 ^h^
Quercetin-3-O-GL	0 ± 0 ^a^	35.9 ± 0.0 ^b^	40.1 ± 0.0 ^b^	38.2 ± 0.0 ^b^	36.1 ± 0.1 ^b^	36.0 ± 0 ^b^	39.3 ± 0.0 ^b^	38.6 ± 0.0 ^b^
Quercetin-3-O-pentosyl-7-O-hexoside	0 ± 0 ^a^	0 ± 0 ^a^	0 ± 0 ^a^	0 ± 0 ^a^	0 ± 0 ^a^	0 ± 0 ^a^	64.0 ± 0.6 ^c^	60.5 ± 0.3 ^b^
Kaempferol-3-O-RU	48.4 ± 0.3 ^e^	44.7 ± 0.1 ^d^	38.4 ± 0.0 ^c^	37.7 ± 0.1 ^a^	37.9 ± 0.0 ^b^	37.6 ± 0.1 ^a^	183.0 ± 8.0 ^f^	190.0 ± 0.0 ^g^
Kaempferol-O-GL	44.8 ± 0.2 ^d^	48.4 ± 0.1 ^e^	36.0 ± 0 ^b^	35.8 ± 0.1 ^b^	0 ± 0 ^a^	0 ± 0 ^a^	38.6 ± 0.1 ^c^	38.4 ± 0.0 ^c^
Isorhamnetin-3-O-RU	0 ± 0 ^a^	0 ± 0 ^a^	0 ± 0 ^a^	34.9 ± 0.0 ^b^	35.2 ± 0.0 ^c^	40.5 ± 0.0 ^e^	0 ± 0 ^a^	35.5 ± 0.0 ^d^
Myricetin-O-RU	0 ± 0 ^a^	0 ± 0 ^a^	42.2 ± 0.1 ^d^	42.3 ± 0.2 ^d^	36.5 ± 0.1 ^b,c^	36.2 ± 0.0 ^b^	0 ± 0 ^a^	36.6 ± 0.1 ^c^
TGL	93.2 ± 0.5 ^a^	129.0 ± 0.0 ^b^	157.0 ± 0.0 ^e^	189.0 ± 0.0 ^f^	146.0 ± 0.0 ^c^	150.0 ± 0.0 ^d^	325.0 ± 7.0 ^g^	399.0 ± 0.0 ^h^
TPC	1132 ± 17 ^c^	1085 ± 0 ^b^	7028 ± 173 ^g^	6851 ± 29 ^f^	1310 ± 35 ^d^	757.0 ± 2.0 ^a^	6014 ± 97 ^e^	6156 ± 73 ^e^

Results are presented as mean ± standard deviation (*n* = 2). Abbreviations: GL—glucoside, RU—rutinoside, TPA—total phenolic acids, TAG—total flavonoid aglycones, TGL—total glycosides, TPC—total phenolic content. Different letters within the same row (a–h), specific to each herb type, indicate significant differences (Duncan test, *p* < 0.05).

**Table 3:** Phenolic profiles of herbal extracts obtained using three DESs (results expressed in mg/100 g).

Herb	Chamomile			Lemon Balm			Nettle			Spearmint		
	DES1	DES2	DES3	DES1	DES2	DES3	DES1	DES2	DES3	DES1	DES2	DES3
Benzoic acid	0 ± 0 ^a^	0 ± 0 ^a^	0 ± 0 ^a^	0 ± 0 ^a^	0 ± 0 ^a^	0 ± 0 ^a^	0 ± 0 ^a^	0 ± 0 ^a^	0 ± 0 ^a^	0 ± 0 ^a^	0 ± 0 ^a^	0 ± 0 ^a^
Caffeic acid	0 ± 0 ^a^	0 ± 0 ^a^	0 ± 0 ^a^	0 ± 0 ^a^	0 ± 0 ^a^	0 ± 0 ^a^	0 ± 0 ^a^	0 ± 0 ^a^	0 ± 0 ^a^	29.8 ± 0.1 ^b^	0 ± 0 ^a^	0 ± 0 ^a^
Chlorogenic acid	29.7 ± 1.0 ^f^	5.6 ± 0.1 ^c^	0 ± 0 ^a^	22.4 ± 0.7 ^e^	0 ± 0 ^a^	0 ± 0 ^a^	17.3 ± 0.2 ^d^	0 ± 0 ^a^	0 ± 0 ^a^	3.7 ± 0.2 ^b^	0 ± 0 ^a^	0 ± 0 ^a^
p-Coumaric acid	0 ± 0 ^a^	0 ± 0 ^a^	0 ± 0 ^a^	0 ± 0 ^a^	0 ± 0 ^a^	0 ± 0 ^a^	0 ± 0 ^a^	0 ± 0 ^a^	0 ± 0 ^a^	0 ± 0 ^a^	0 ± 0 ^a^	0 ± 0 ^a^
Ellagic acid	34.2 ± 1.0 ^f^	6.2 ± 0.1 ^c^	5.1 ± 0.3 ^b^	5.4 ± 0.1 ^b^	0 ± 0 ^a^	0 ± 0 ^a^	13.9 ± 1.0 ^e^	0 ± 0 ^a^	0 ± 0 ^a^	221.0 ± 3.0 ^g^	15.5 ± 0.5 ^e^	9.6 ± 0.5 ^d^
Ferulic acid	16.4 ± 0.1 ^d^	15.8 ± 0.0 ^c^	15.4 ± 0.0 ^b^	0 ± 0 ^a^	0 ± 0 ^a^	0 ± 0 ^a^	0 ± 0 ^a^	0 ± 0 ^a^	0 ± 0 ^a^	15.8 ± 0.0 ^c^	0 ± 0 ^a^	0 ± 0 ^a^
Gallic acid	249.0 ± 7.0 ^d^	0 ± 0 ^a^	0 ± 0 ^a^	151.0 ± 3 ^b^	0 ± 0 ^a^	0 ± 0 ^a^	232.0 ± 10 ^c^	0 ± 0 ^a^	0 ± 0 ^a^	0 ± 0 ^a^	0 ± 0 ^a^	0 ± 0 ^a^
Gentisic acid	0 ± 0 ^a^	0 ± 0 ^a^	0 ± 0 ^a^	0 ± 0 ^a^	0 ± 0 ^a^	0 ± 0 ^a^	0 ± 0 ^a^	0 ± 0 ^a^	0 ± 0 ^a^	37.8 ± 0.2 ^b^	0 ± 0 ^a^	0 ± 0 ^a^
Rosmarinic acid	39.2 ± 0.8 ^f^	28.0 ± 0.1 ^d^	21.5 ± 0.1 ^c^	259.0 ± 7.0 ^i^	59.6 ± 0.4 ^h^	43.6 ± 0.3 ^g^	20.3 ± 0.1 ^b^	19.2 ± 0.0 ^b^	0 ± 0 ^a^	461.0 ± 9.0 ^j^	35.0 ± 0.2 ^e^	36.7 ± 0.3 ^f^
Salicylic acid	0 ± 0 ^a^	0 ± 0 ^a^	0 ± 0 ^a^	2.6 ± 0.0 ^b^	0 ± 0 ^a^	0 ± 0 ^a^	0 ± 0 ^a^	0 ± 0 ^a^	0 ± 0 ^a^	14.3 ± 0.5 ^c^	0 ± 0 ^a^	0 ± 0 ^a^
Syringic acid	2.4 ± 0.0 ^b^	3.8 ± 0.1 ^c^	2.4 ± 0.1 ^b^	0 ± 0 ^a^	0 ± 0 ^a^	0 ± 0 ^a^	0 ± 0 ^a^	0 ± 0 ^a^	0 ± 0 ^a^	3.8 ± 0.1 ^c^	0 ± 0 ^a^	0 ± 0 ^a^
Vanillic acid	0 ± 0 ^a^	0 ± 0 ^a^	0 ± 0 ^a^	0 ± 0 ^a^	0 ± 0 ^a^	0 ± 0 ^a^	0 ± 0 ^a^	0 ± 0 ^a^	0 ± 0 ^a^	0 ± 0 ^a^	0 ± 0 ^a^	0 ± 0 ^a^
TPA	370.0 ± 7.0 ^g^	59.3 ± 0.0 ^f^	44.5 ± 0.3 ^c^	440.0 ± 11.0 ^h^	59.6 ± 0.4 ^f^	43.6 ± 0.3 ^c^	284.0 ± 9.0 ^a^	19.2 ± 0.0 ^b^	0 ± 0 ^a^	786.0 ± 7.0 ^i^	50.5 ± 0.7 ^e^	46.3 ± 0.2 ^d^
Apigenin	10.6 ± 0.2 ^e^	7.0 ± 0.1 ^d^	4.9 ± 0.0 ^c^	0 ± 0 ^a^	0 ± 0 ^a^	0 ± 0 ^a^	0 ± 0 ^a^	0 ± 0 ^a^	0 ± 0 ^a^	7.8 ± 0.1 ^d^	1.4 ± 0.0 ^b^	1.3 ± 0.0 ^b^
(+)-Catechin	0 ± 0 ^a^	0 ± 0 ^a^	0 ± 0 ^a^	0 ± 0 ^a^	0 ± 0 ^a^	0 ± 0 ^a^	0 ± 0 ^a^	0 ± 0 ^a^	0 ± 0 ^a^	7.7 ± 0.0 ^b^	0 ± 0 ^a^	0 ± 0 ^a^
Kaempferol	23.6 ± 0.1 ^e^	18.4 ± 0.1 ^d^	17.8 ± 0.0 ^c^	17.4 ± 0.0 ^b^	0 ± 0 ^a^	0 ± 0 ^a^	17.3 ± 0.0 ^b^	0 ± 0 ^a^	0 ± 0 ^a^	28.7 ± 0.1 ^f^	17.7 ± 0.0 ^c^	17.5 ± 0.0 ^b^
Myricetin	0 ± 0 ^a^	0 ± 0 ^a^	0 ± 0 ^a^	0 ± 0 ^a^	0 ± 0 ^a^	0 ± 0 ^a^	0 ± 0 ^a^	0 ± 0 ^a^	0 ± 0 ^a^	0 ± 0 ^a^	0 ± 0 ^a^	0 ± 0 ^a^
Naringenin	15.3 ± 0.0 ^b^	15.2 ± 0.0 ^b^	15.1 ± 0.0 ^b^	0 ± 0 ^a^	0 ± 0 ^a^	0 ± 0 ^a^	0 ± 0 ^a^	0 ± 0 ^a^	0 ± 0 ^a^	16.0 ± 0.0 ^c^	15.1 ± 0.0 ^b^	15.1 ± 0.0 ^b^
Quercetin	19.3 ± 0.0 ^e^	17.3 ± 0.0 ^b^	17.7 ± 0.0 ^c^	17.4 ± 0.0 ^b^	0 ± 0 ^a^	17.7 ± 0.0 ^c^	17.7 ± 0.0 ^c^	17.8 ± 0.0 ^c^	17.9 ± 0.0 ^d^	0 ± 0 ^a^	0 ± 0 ^a^	18.0 ± 0.0 ^d^
Rutin	5.6 ± 0.0 ^e^	5.2 ± 0.0 ^d^	4.7 ± 0.0 ^b^	4.9 ± 0.0 ^b^	0 ± 0 ^a^	0 ± 0 ^a^	13.3 ± 0.2 ^h^	5.9 ± 0.0 ^e^	5.0 ± 0.0 ^c^	32.1 ± 0.3 ^i^	6.8 ± 0.0 ^f^	7.2 ± 0.1 ^g^
TAG	74.4 ± 0.4 ^i^	63.2 ± 0.0 ^h^	60.2 ± 0.0 ^g^	39.7 ± 0.0 ^d^	0 ± 0 ^a^	17.7 ± 0.0 ^b^	48.3 ± 0.2 ^f^	23.6 ± 0.1 ^c^	22.9 ± 0.0 ^c^	92.3 ± 0.6 ^j^	41.1 ± 0.0 ^e^	59.2 ± 0.1 ^g^
Quercetin-3-O-GL	0 ± 0 ^a^	0 ± 0 ^a^	0 ± 0 ^a^	17.9 ± 0.0 ^b^	0 ± 0 ^a^	0 ± 0 ^a^	0 ± 0 ^a^	0 ± 0 ^a^	0 ± 0 ^a^	20.5 ± 0.1 ^c^	0 ± 0 ^a^	0 ± 0 ^a^
Quercetin-3-O-pentosyl-7-O-hexoside	0 ± 0 ^a^	0 ± 0 ^a^	0 ± 0 ^a^	0 ± 0 ^a^	0 ± 0 ^a^	0 ± 0 ^a^	0 ± 0 ^a^	0 ± 0 ^a^	0 ± 0 ^a^	22.7 ± 0.1 ^c^	17.4 ± 0.0 ^b^	17.6 ± 0.0 ^b^
Kaempferol-3-O-RU	18.0 ± 0.0 ^b^	0 ± 0 ^a^	0 ± 0 ^a^	0 ± 0 ^a^	0 ± 0 ^a^	0 ± 0 ^a^	0 ± 0 ^a^	0 ± 0 ^a^	0 ± 0 ^a^	53.6 ± 0.1 ^d^	19.6 ± 0.0 ^c^	19.6 ± 0.1 ^c^
Kaempferol-O-GL	18.6 ± 0.1 ^e^	17.8 ± 0.0 ^c^	17.7 ± 0.0 ^c^	0 ± 0 ^a^	0 ± 0 ^a^	0 ± 0 ^a^	0 ± 0 ^a^	0 ± 0 ^a^	0 ± 0 ^a^	18.1 ± 0.0 ^d^	17.1 ± 0.0 ^b^	0 ± 0 ^a^
Isorhamnetin-3-O-RU	0 ± 0 ^a^	0 ± 0 ^a^	0 ± 0 ^a^	0 ± 0 ^a^	0 ± 0 ^a^	0 ± 0 ^a^	0 ± 0 ^a^	0 ± 0 ^a^	0 ± 0 ^a^	0 ± 0 ^a^	0 ± 0 ^a^	0 ± 0 ^a^
Myricetin-O-RU	0 ± 0 ^a^	0 ± 0 ^a^	0 ± 0 ^a^	0 ± 0 ^a^	0 ± 0 ^a^	0 ± 0 ^a^	0 ± 0 ^a^	0 ± 0 ^a^	0 ± 0 ^a^	0 ± 0 ^a^	0 ± 0 ^a^	0 ± 0 ^a^
TGL	36.6 ± 0.1 ^c^	17.8 ± 0.0 ^b^	17.7 ± 0.0 ^b^	17.9 ± 0.0 ^b^	0 ± 0 ^a^	0 ± 0 ^a^	0 ± 0 ^a^	0 ± 0 ^a^	0 ± 0 ^a^	115.0 ± 0.0 ^f^	54.1 ± 0.0 ^e^	37.2 ± 0.0 ^d^
TPC	481.0 ± 7.0 ^j^	140.3 ± 0.0 ^f^	122.4 ± 0.0 ^e^	497.6 ± 11.0 ^k^	59.6 ± 0.4 ^c^	61.3 ± 0.3 ^d^	332.3 ± 10.0 ^i^	42.8 ± 0.0 ^b^	22.9 ± 0.0 ^a^	993.3 ± 6.0 ^l^	145.7 ± 1.0 ^h^	142.7 ± 0.0 ^g^

Results are presented as mean ± standard deviation (*n* = 2). Abbreviations: DES1—choline chloride:malonic acid (ChCl:MalA) (1:1), DES2—choline chloride:glycerol (ChCl:Gly) (1:2), DES3—choline chloride:glucose (ChCl:Glu) (2:1), GL—glucoside, RU—rutinoside, TPA—total phenolic acids, TAG—total flavonoid aglycones, TGL—total glycosides, TPC—total phenolic content. Different letters within the same row (a–l), specific to each herb type, indicate significant differences (Duncan test, *p* < 0.05).

**Table 4:** Contents of elements in herbs.

Element	Concentration * ± SD (mg/kg)			
	Chamomile	Lemon Balm	Nettle	Spearmint
Na	843 ± 35 ^d^	84.3 ± 3.8 ^b^	37.3 ± 3.0 ^a^	332 ± 12 ^c^
K	16,597 ± 210 ^a^	23,229 ± 964 ^b^	23,644 ± 1112 ^b^	24,979 ± 710 ^c^
Ca	7065 ± 143 ^a^	14,975 ± 792 ^b^	32,926 ± 1210 ^c^	14,325 ± 958 ^b^
Mg	1461 ± 49 ^a^	3956 ± 144 ^b^	4778 ± 169 ^c^	5354 ± 180 ^d^
Mn	38.8 ± 1.0 ^a^	76.7 ± 2.0 ^c^	69.1 ± 3.5 ^b^	152 ± 4 ^d^
Zn	14.6 ± 0.3 ^a^	31.5 ± 0.6 ^c^	21.4 ± 0.7 ^b^	31.7 ± 0.8 ^c^
Fe	105 ± 4 ^a^	495 ± 14 ^d^	312 ± 11 ^b^	383 ± 13 ^c^
Cu	4.5 ± 0.1 ^a^	8.6 ± 0.4 ^b^	13.6 ± 0.1 ^c^	13.3 ± 0.4 ^c^
Cd	0.506 ± 0.013 ^d^	0.088 ± 0.008 ^c^	0.046 ± 0.005 ^a^	0.064 ± 0.003 ^b^

* *n* = 6; SD—standard deviation; Different letters within the same row (a–d) indicate significant differences between concentrations of each element determined in four herbs (Duncan test, *p* < 0.05).

**Table 5:** Operating Conditions of the ICP-MS Spectrometer.

Parameter	Data
RF Power (W)	1550
Spray Chamber Temperature (°C)	2
Sampling Depth (mm)	10.0
Plasma Gas (L/min)	15.0
Auxiliary Gas (L/min)	0.90
Nebulizer Gas Flow (L/min)	1.01
Lens Tune	Autotune
Collision Gas Flow He (mL/min)	5.0
Energy Discrimination (mV)	4.2
Isotopes Measured (Internal Standard Isotopes)	^23^Na (^73^Ge), ^39^K (^45^Sc), ^44^Ca (^45^Sc), ^24^Mg (^89^Y), ^55^Mn (^115^In), ^66^Zn (^115^In), ^56^Fe (^73^Ge), ^63^Cu (^115^In), ^111^Cd (^115^In)
Integration Time (s)	0.3

## Data Availability

The data presented in this study are available on request from the corresponding author.
